# Effect of four *ABCB1* genetic polymorphisms on the accumulation of darunavir in HEK293 recombinant cell lines

**DOI:** 10.1038/s41598-021-88365-7

**Published:** 2021-04-26

**Authors:** Gabriel Stillemans, Happy Phanio Djokoto, Kévin-Alexandre Delongie, Halima El-Hamdaoui, Nadtha Panin, Vincent Haufroid, Laure Elens

**Affiliations:** 1 Integrated PharmacoMetrics, PharmacoGenomics and PharmacoKinetics, Louvain Drug Research Institute, Université Catholique de Louvain, Brussels, Belgium; 2 Louvain Centre for Toxicology and Applied Pharmacology, Institut de Recherche Expérimentale et Clinique, Université Catholique de Louvain, Brussels, Belgium; 3 Department of Clinical Chemistry, Cliniques Universitaires Saint-Luc, Brussels, Belgium

**Keywords:** Immunological deficiency syndromes, Genetics research, Pharmacogenetics

## Abstract

The intracellular penetration of darunavir, a second-generation HIV protease inhibitor, is limited by the activity of the efflux P-glycoprotein (ABCB1). ABCB1 expression and/or activity levels can vary between individuals due to genetic polymorphisms including the c.1199G>A, c.1236C>T, c.2677G>T and c.3435C>T variants, which could in part explain why the pharmacokinetics of darunavir are so variable from one individual to another. While a few clinical studies have failed to demonstrate an influence of these polymorphisms on darunavir pharmacokinetics, drug-drug interactions and methodological limitations may have prevented them from revealing the true influence of *ABCB1* variants. In this work, we report on the intracellular accumulation of darunavir in recombinant HEK293 cell lines expressing wild-type ABCB1 or one of several variants: ABCB1 1199A, ABCB1 3435T, and ABCB1 1236T/2677T/3435T. We demonstrate that while ABCB1 expression limits intracellular accumulation of darunavir, there is no significant difference in efflux activity between cells expressing wild-type ABCB1 and those that express any of the studied variants.

## Introduction

Darunavir (DRV) is a second-generation protease inhibitor used as part of a multi-drug cocktail in HIV therapy^1,2^. Its pharmacokinetics (PK) are known to be highly heterogeneous, with the plasma AUC varying more than fivefold between individuals receiving the same dosing regimen^3^. Many individual factors could potentially contribute to this variability, including genetic variations in key biotransformation enzymes and transporters involved in the disposition of DRV. As far as transporters are concerned, DRV is a substrate of influx transporters of the SLCO family (SLCO1A2, SLCO1B1 and SLCO1B3) and efflux transporters of the ABC family (ABCB1, ABCC2)^4–6^. The *ABCB1* gene, which encodes the P-glycoprotein, is of particular interest^7^. ABCB1 efflux limits drug penetration in enterocytes, therefore decreasing the oral bioavailability of DRV, which is low when administered alone^8^. This is one of the reasons why DRV is always coadministered with a PK booster such as ritonavir (RTV) or cobicistat (COB), which reduces first-pass metabolism and systemic clearance of DRV, mainly through inhibition of CYP metabolism and ABCB1 activity. ABCB1 also controls drug accumulation in lymphocytes, the active site of antiretrovirals, thereby directly limiting the ability of DRV to reach its target. It is also expressed in other organs, including the blood–brain barrier, meaning it could limit penetration in the central nervous system, which has been described as a sanctuary site for HIV. And while single nucleotide polymorphisms (SNPs) that alter the expression and/or activity of *ABCB1* have been described and could potentially explain—at least in part—the important PK variability that characterizes DRV, their exact contribution remains unclear. Functional *ABCB1* SNPs of interest include *ABCB1* c.1199G>A, c.1236C>T, c.2677T>G/A and c.3435C>T (Table 1). Both *ABCB1* c.1236C>T and c.2677T>G/A are in strong linkage disequilibrium with c.3435C>T, and the haplotype they define is considered to capture most of the genetic variability for *ABCB1*^9^. There is some controversy over the impact of these variants on the PK of several drugs, perhaps due to differences in studied populations and experimental protocols, but also due to substrate-specific effects, which explains why observations made for one drug cannot be directly transposed to another, even if they are both known substrates of this efflux transporter. Moreover, even when in vitro associations are found, these do not necessarily translate into in vivo associations probably due to compensatory mechanisms, confounding factors or DDIs^10^.

**Table 1:** *ABCB1* polymorphisms of interest.

Nucleotide change	rs number	Description	MAF		
			Euro (%)	Asian (%)	African (%)
c.1199G>A	rs2229109	Missense variant: Ser400Asn. Effect are possibly substrate-dependent	3	< 1	< 1
c.1236C>T	rs1128503	Synonymous variant. Controversial effects	42	59–63	14
c.2677T>G/A	rs2032582	Missense variant: Ser893Ala/Thr. Controversial effects	41	40–59	3
c.3435C>T	rs1045642	Synonymous variant. Alters the timing of protein folding and decreases mRNA stability. Effect are possibly substrate-dependent	52	40–58	15

*Euro* European, *MAF* minor allele frequency. MAFs are from the 1000 Genomes Project^11^. Asian MAFs include South and East Asians.

The influence of some of these *ABCB1* variants on DRV PK has been investigated in clinical studies^3,12–14^, but they did not appear to be good predictors of inter-individual variability. However, the inhibitory effect of RTV and COB on ABCB1 could limit the in vivo effect of genetic variants for this transporter towards DRV^6^. Combined with small sample sizes and other confounding factors, this makes it difficult to delineate the true contribution of *ABCB1* variants. Therefore, we decided to study the intracellular accumulation of DRV in human embryonic kidney cells (HEK293) overexpressing the wild-type ABCB1 or one of several variants (ABCB1 1199A, ABCB1 3435T, and ABCB1 1236T/2677T/3435T) to clarify this point.

## Methods

### Chemicals and reagents

Dulbecco’s Modified Eagle Medium (DMEM), fetal bovine serum (FBS), penicillin/streptomycin and enzyme-free cell dissociation buffer were purchased from Gibco (Thermo Fisher); G418 from Roche; flow cytometry antibodies from BD Biosciences (FITC mouse anti-human CD243, clone 17F9, reference 557002; and FITC mouse IgG2b κ isotype control, clone 27–35, reference 555742); DRV, deuterated DRV (DRV-d9) and COB from Toronto Research Chemicals. All chemicals used in drug quantification were of analytical grade.

### Characterization of cell lines

The generation and characterization of recombinant cell lines have been described in previous work conducted by our lab^15,16^. Briefly, HEK293 cells were transfected with plasmids carrying wild-type *ABCB1* (1199G-1236C-2677G-3435C, HEK_WT_); variants 1199G-1236C-2677G-3435T (HEK_CGT_), 1199G-1236T-2677T-3435T (HEK_TTT_) or 1199A-1236C-2677G-3435C (HEK_1199A_) obtained using site-directed mutagenesis; or an empty vector (HEK_control_). HEK293 cells feature low endogenous levels of expression of ABCB1, ensuring that all ABCB1 expression originates from the transfection process. This model has previously been extensively characterized using flow cytometry, Western blot, and fluorescence microscopy, and validated using reference substrates and inhibitors of ABCB1^15,16^. Since the same cells were used, only flow cytometry was used to re-characterize them. After thawing, cells were grown in DMEM, FBS 10% and penicillin/streptomycin 1% at 37 °C in the presence of 5% CO_2_. After at least seven days of growth in the presence of the selection antibiotic G418 (1 g/l), ABCB1 expression was assessed by flow cytometry: 0.5 × 10^6^ cells were collected by centrifugation and washed twice with ice-cold buffer (PBS, FBS 1%, EDTA 1 mM) then re-suspended in buffer supplemented with 10% anti-ABCB1 antibody, 10% isotype control or in buffer with no antibody, and left to incubate for 45 min on ice and in the dark. Finally, the cells were washed with buffer and resuspended before being analyzed using a BD FACSVerse flow cytometer (for characterization) or BD FACSAria III (for cell sorting) and the BD FACSuite software. Additional data analysis and plotting were carried out in FlowJo (version 10.6.1).

### Intracellular accumulation experiments

0.35 × 10^6^ cells were seeded on poly-l-lysine-coated 24-well plates and incubated overnight. The next day, DRV dilutions in DMEM were prepared from a stock solution and added in each well at a final concentration of 0.5, 1, 2.5, 5 or 10 mg/l (final volume: 500 µl). These values were chosen to cover the range of total plasma concentrations found in patients treated with COB- or RTV-boosted DRV. Cells were incubated in triplicate at 37 °C in the presence of 5% CO_2_ for 2 h. The plates were then centrifuged for 5 min at 450×*g*, 4 °C, then kept on ice for the remainder of the experiment to block drug efflux. Cells were washed twice with 500 µl of ice-cold PBS, then 400 µl of a mixture of methanol/water 60/40% (v/v) containing 20 ng/ml DRV-d9 was added and cells were detached by scratching the surface of the well. Cell suspensions were kept at − 20 °C until quantification.

### Cell viability

The viability of HEK cells in the presence of increasing concentrations of DRV (1, 5 and 10 mg/l) was assessed using the WST-1 assay according to the manufacturer’s instructions. Cells were grown in 96-well plates and viability was assessed separately for the HEK_control_ and HEK_WT_ groups. Absorbance was measured at 450 nm after 0.5, 1, 1.25, 1.5 and 1.75 h of incubation.

### Drug quantification

DRV intracellular concentrations were determined using an adapted version of a previously published liquid chromatography-tandem mass spectrometry (LC–MS/MS) method^17^. Cell suspensions were vortex mixed, sonicated for 5 min, placed on an orbital shaker for 2 h and centrifuged for 10 min at 10,500×*g*. The supernatant was transferred to a vial for injection and the pellet was set aside for protein quantification. Calibrators ranging from 1.25 to 125 ng/ml were prepared in a similar fashion. Chromatographic separation was achieved on a Waters UPLC BEH C18 1.7 µm column (2.1 × 50 mm) maintained at 40 °C. The injection volume was 5 µl and the flow rate was 0.5 ml/min. The mobile phase consisted of a gradient of water/formic acid 0.1% (mobile phase A) and acetonitrile (mobile phase B), starting with 95% A and 5% B, ramping up to 80% B over 6 min, then returning to 5% B at 6.1 min and remaining at this ratio until the end of the run (total run time: 8 min). The MS system was a Xevo TQS-micro tandem quadrupole mass spectrometer (Waters). The following ion transitions were monitored: 548.2 > 392.3 for DRV and 557.3 > 113 for DRV-d9.

### Protein quantification

Total proteins were quantified in pellets using the BCA kit (Thermofisher Scientific) according to the manufacturer’s instructions. Absolute DRV concentrations were normalized by total protein content.

### Statistical analysis

Each set of experiments were performed thrice. Normalized intracellular concentrations at each dose level were compared using one-way ANOVA (α = 0.05) followed by Tukey’s HSD test. All statistical analyses were carried out in R (version 3.6.3)^18^.

## Results

### Flow cytometry

**Figure 1:**
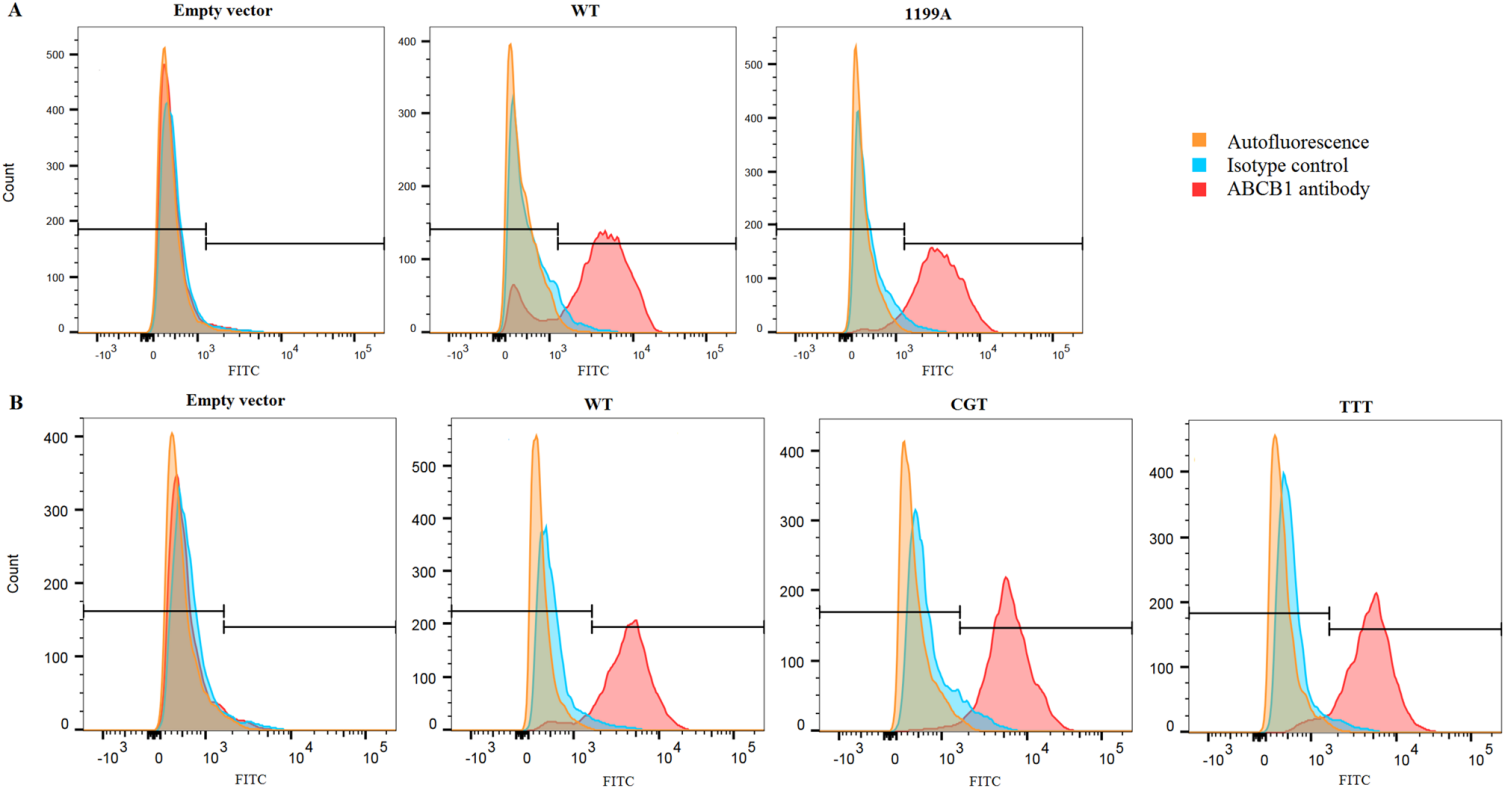
ABCB1 expression assessed by flow cytometry for cells stained with ABCB1 antibody, isotype control and no staining (autofluorescence). (**A**) Histograms of HEK_control_, HEK_WT_ and HEK_1199A_. (**B**) Histograms of HEK_control_, HEK_WT_, HEK_CGT_ and HEK_TTT_. *FITC* Fluorescein isothiocyanate.

ABCB1 expression in our model was confirmed by flow cytometry analysis. After sorting, all cell lines expressed comparable levels of ABCB1 (> 80%) except for HEK_control_, which, predictably, were characterized by low levels of expression (Fig. 1).

### Intracellular accumulation

**Figure 2:**
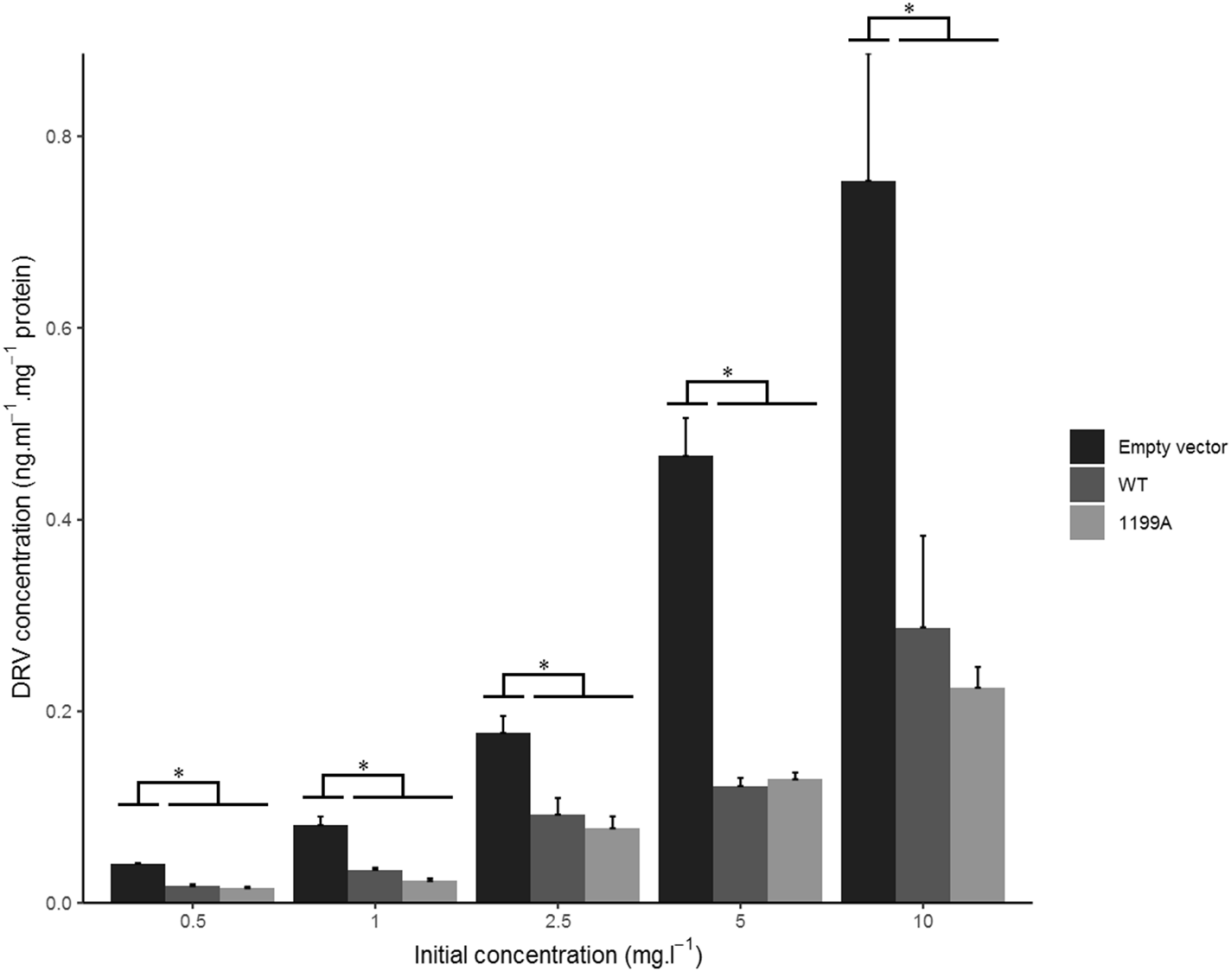
Intracellular protein-normalized DRV concentrations in HEK_control_ (empty vector), HEK_WT_ and HEK_1199A_ cells at several dose levels after 2 h of incubation. Results reported as mean + standard error (n = 3). *Denotes statistically significant difference (p < 0.05) between groups.

**Figure 3:**
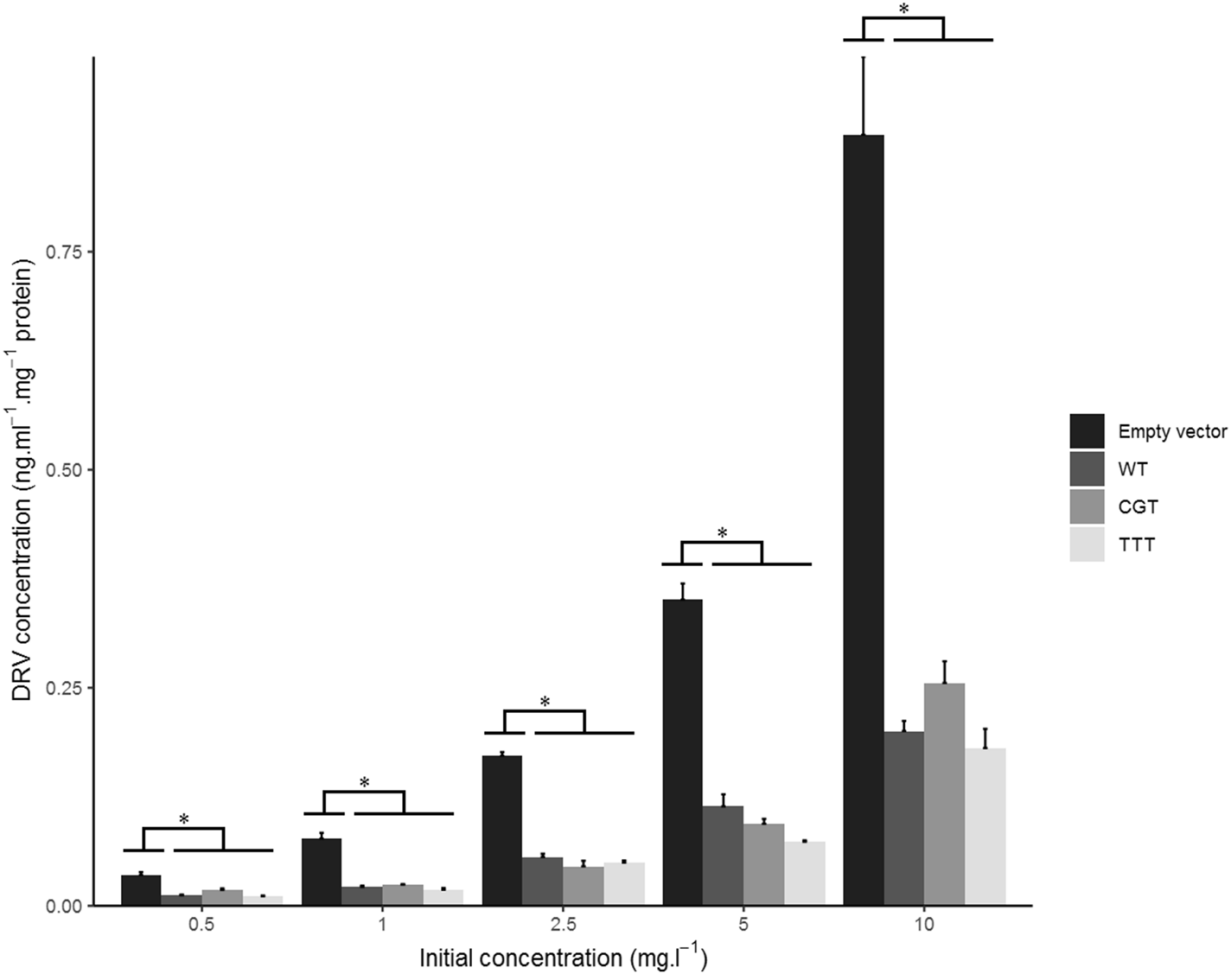
Intracellular protein-normalized DRV concentrations in HEK_control_ (empty vector), HEK_WT*,*_ HEK_CGT_ and HEK_TTT_ cells at several dose levels after 2 h of incubation. Results reported as mean + standard error (n = 3). *Denotes statistically significant difference (p < 0.05) between groups.

Intracellular concentrations of DRV, adjusted by the total protein content of the sample, markedly differed between cell lines: they were significantly lower in all ABCB1-expressing cells compared to control cells transfected with an empty plasmid in all experiments (Figs. 2 and 3), confirming that DRV is a substrate of ABCB1 and that drug efflux greatly limits cellular penetration in this model. There was no difference in intracellular concentrations between cells expressing the c.1199G or the c.1199A variant (p = 0.89, 0.98, 0.81, 0.38, 0.56 at the 10, 5, 2.5, 1 and 0.5 mg.l^-1^ dose levels, respectively) (Fig. 2). Likewise, concerning the *ABCB1* haplotype defined by the c.1236C>T, c.2677G>T and c.3435C>T variants, intracellular concentrations did not significantly differ between CGC (wild-type) versus CGT cells (p = 0.85, 0.66, 0.37, 0.95, 0.20), CGC versus TTT (p = 0.99, 0.16, 0.74, 0.93, 0.98), or CGT versus TTT (p = 0.7, 0.64, 0.9, 0.68, 0.12) (Fig. 3).

### Cell viability

**Figure 4:**
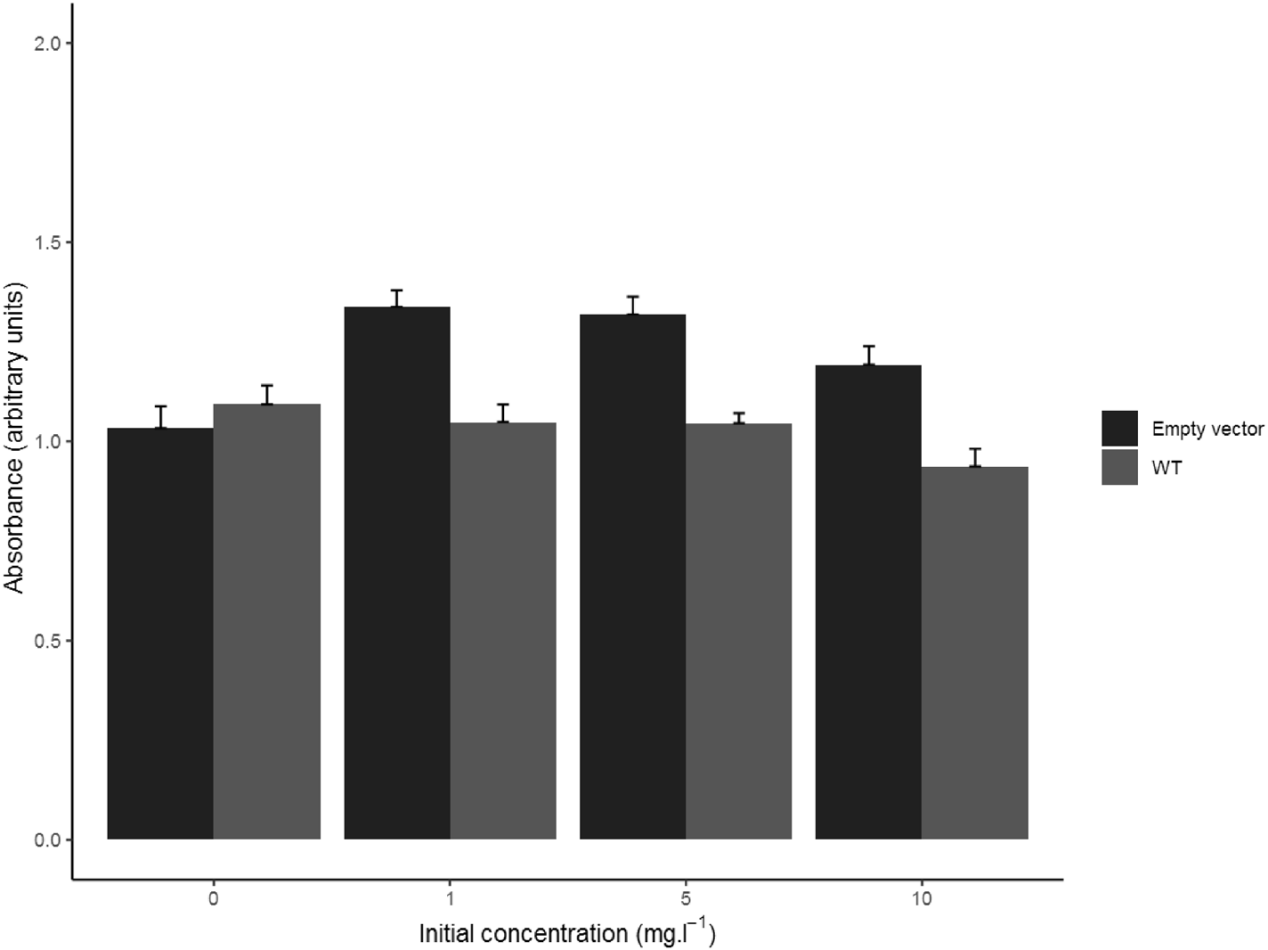
WST-1 cell viability assay results in HEK_control_ (empty vector) and HEK_WT_ cells at several dose levels (0 being the control condition) after 1.75 h of incubation. Results reported as mean absorbance + standard error (n = 6).

Cell viability was unaffected by DRV at concentration levels ranging from 1 to 10 mg/l. The results of these experiments are displayed in Fig. 4.

## Discussion

Several pharmacogenetic studies have attempted to determine the influence of *ABCB1* variants on DRV pharmacokinetics. Calcagno et al*.* studied the paired PK of DRV in plasma and cerebrospinal fluid (CSF) in 41 patients and showed that there was no influence of *ABCB1* c.1236C>T, c.2677G>T or c.3435C>T on DRV penetration in the central nervous system, as assessed by the ratio of concentrations between the CSF and plasma^14^. Moltó et al*.* developed a population PK model of DRV on 75 subjects and assessed the influence of 148 SNPs in 15 genes, including ABCB1 c.1236C>T and c.2677G>T, neither of which showed a significant effect on PK parameters^13^. PK parameters were similarly unaffected by the c.1236C>T and c.3435C>T variants in a small group of 25 patients in another population study^19^. Moreover, Nagano et al*.* found that the *ABCB1* c.3435C>T variant was not correlated with concentrations of DRV in either plasma or peripheral blood mononuclear cells (PBMCs) in a group of 19 patients^12^. Finally, we recently published a population PK model developed on 127 patients for which neither *ABCB1* c.1199G>A nor *ABCB1* c.3435C>T were correlated with DRV PK parameters^3^. Although these studies were not necessarily powered with a specific set of covariates in mind, they suggest that genetic polymorphisms in *ABCB1* either do not alter protein activity toward this molecule, or that other, unidentified factors mask or neuter the effect of *ABCB1* variants in vivo. RTV- or COB-based boosting likely has a role to play since both of these boosters are capable of inhibiting ABCB1 to some degree, but other factors may also contribute. Besides these considerations related to DRV based therapy, there have been conflicting results in the literature regarding the effect of these genetic variants not only in vivo but also in vitro. For instance, Woodahl et al*.* showed that *ABCB1*-mediated efflux for older protease inhibitors such as lopinavir, amprenavir, indinavir, saquinavir and RTV was increased in cells expressing the c.1199A variant compared to the wild-type protein^20^. Meanwhile, tumor resistance to anticancer agents was increased in c.1199A cells for certain agents such as vinblastine, vincristine, paclitaxel and etoposide, with fold-changes ranging from 1.9 to 11 depending on the drug, but was unchanged for doxorubicin^21^. Dessilly et al*.* also showed that the c.1199G>A variant modulated the intracellular accumulation of tacrolimus, but not that of ciclosporine^15^. Further, they showed that the 1236-2677-3435 haplotype had little to no effect on the accumulation and activity of several tyrosine kinase inhibitors (dasatinib, nilotinib and ponatinib), whereas the variant *ABCB1* displayed lower activity toward imatinib^16^. Sennesael et al*.* also showed that there was no effect of any of the four aforementioned SNPs on the efflux of the anticoagulant drug rivaroxaban^22^. Because of these conflicting results, there is a need for case-by-case assessment of drug accumulation to determine the relevance of genetic variants in *ABCB1*. An existing model of intracellular accumulation was used to assess the influence of *ABCB1* variants. Our experiments showed that the c.1199G>A, c.1236C>T, c.2677G>T and c.3435C>T variants have no effect on the ability of ABCB1 to transport DRV in this model, similarly to what was observed for other substrates, such as rivaroxaban. While it could be argued that HEK293 cells are not a physiologically representative model, they feature a low basal expression level of ABCB1, ensuring all ABCB1 activity in our model can be attributed to the transfection process^15,16^, unlike other cell lines like Caco-2, that would be more representative of the digestive tract but would also introduce a bias due to a combination of native and transfection-induced expression. Further, this in vitro model is characterized by supraphysiological levels of ABCB1 expression and these SNPs may exert slightly different effects with in vivo levels. In any event, variants in other influx and efflux transporters, as well as a range of genetic and non-genetic factors, could potentially explain the wide range of plasma and intracellular concentrations that have been reported for DRV. In conclusion, using a recombinant model of HEK293 cells, we showed that *ABCB1* variants do not appear to modulate the ability of this efflux pump to transport DRV, and as such, they do not alter the intracellular accumulation of this antiretroviral.

## Data Availability

Data generated and analyzed during this study are available from the authors on reasonable request.

## Abbreviations

COB: Cobicistat
CSF: Cerebrospinal fluid
DRV: Darunavir
DRV-d9: Deuterated darunavir
EDTA: Ethylenediaminetetraacetate
FBS: Fetal bovine serum
FITC: Fluorescein isothiocyanate
HEK: Human embryonic kidney
LC–MS/MS: Liquid chromatography coupled with tandem mass spectrometry
MAF: Minor allele frequency
PBS: Phosphate buffer saline
PK: Pharmacokinetic
RTV: Ritonavir
SNP: Single nucleotide polymorphism
